# Preclinical Comparison of Distal Off-Pump Anastomotic Remodeling: Hand-Sewn Versus ELANA Heart Bypass

**DOI:** 10.1177/15569845221079606

**Published:** 2022-03-17

**Authors:** David Stecher, Marieke Hoogewerf, Bart P. van Putte, Shadan Osman, Pieter A. Doevendans, Cornelis Tulleken, Lex van Herwerden, Gerard Pasterkamp, Marc P. Buijsrogge

**Affiliations:** 1 Department of Cardiothoracic Surgery, University Medical Center Utrecht, The Netherlands; 2 Department of Cardiology, University Medical Center Utrecht, The Netherlands; 3 Department of Cardiothoracic Surgery, St Antonius Hospital, Nieuwegein, The Netherlands; 4 Department of Cardiothoracic Surgery, Academic Medical Center, Amsterdam, The Netherlands; 5 Netherlands Heart Institute, Utrecht, The Netherlands; 6 Department of Neurosurgery, University Medical Center Utrecht, The Netherlands; 7 Department of Experimental Cardiology, University Medical Center Utrecht, The Netherlands

**Keywords:** coronary artery bypass grafting, OPCAB, sutureless anastomosis, coronary connector, coronary revascularization

## Abstract

**Objective:** The ELANA Heart Bypass System is a new sutureless technique for coronary anastomoses. A titanium clip connects the graft with the coronary artery, whereafter the arteriotomy is performed by excimer laser. Since this anastomotic construction evidently differs from the standard hand-sewn anastomosis, we aim to evaluate the process of anastomotic healing and remodeling. **Methods:** Preclinical evaluation of anastomotic remodeling in 42 pigs who underwent off-pump left internal mammary artery to left anterior descending artery anastomosis by either the ELANA Heart Bypass (*n* = 24) or the hand-sewn (*n* = 18) technique. Anastomotic remodeling was evaluated by scanning electron microscopy and histology in short-term follow-up intervals up to 3 months. Anastomotic patency is determined by coronary angiography at latest follow-up before termination. **Results:** The nonendothelial surface of both the ELANA and the hand-sewn anastomoses were covered with neointima from 14 days onwards. Only half the amount of intima hyperplasia was present in the anastomotic surface of the patent ELANA anastomosis, compared with the hand-sewn anastomosis (98 [48–1358] vs 218 [108–296] µm, *P* = 0.001). Yet patency of the ELANA was inferior to the hand-sewn anastomoses (79% vs 100%, *P* = 0.06). **Conclusions:** This study shows the technical perioperative feasibility of the ELANA Heart Bypass System. Although limited intima hyperplasia was observed, hand-sewn anastomoses had superior patency during follow-up. The results of this trial suggest that an additional study with a new prototype is required before clinical implementation.

**Central Message**

The ELANA Heart Bypass System is a sutureless technique for coronary anastomoses. This study found that remodeling of the ELANA anastomosis was as good as the hand- sewn anastomosis. A smaller and lighter device could improve patency rates.

## Introduction

The 2018 European Society of Cardiology and European Association for Cardio-Thoracic Surgery guidelines on myocardial revascularization recommend coronary artery bypass grafting (CABG) for patients suffering from left main stenosis, proximal left anterior descending artery (LAD) stenosis, or triple-vessel disease with high SYNTAX scores and/or diabetes mellitus.^1^ The conventional method for the coronary anastomosis is the hand-sewn technique, which is most widely performed by a running suture with a monofilament wire. Patency rates vary from 91% for internal mammary artery (IMA) and 80% to 85% for venous grafts 6 to 12 months postoperatively, to 51% to 98% for IMA and 58% to 83% for venous grafts >4 years postoperatively.^2,3^

Despite the satisfying results, CABG remains an invasive procedure with substantial impact on patient quality of life in the first period after surgery. Furthermore, there is a continuous need for standardization and simplification to reduce the risk of human errors. From these perspectives, the ELANA Heart Bypass technique has been developed.^5,6^ This technique enables a sutureless distal anastomosis in off-pump CABG by connecting donor graft and coronary artery per implant clip and performing arteriotomy thereafter per excimer (contact) laser. Whereas the technique was originally described for neurosurgical approaches, the first cardiovascular prototypes suited large caliber coronary arteries (3.0 mm).^5,7–9^ Following multiple device updates, the ELANA Heart Bypass ultimately fits 1.5 mm coronary targets.^5^ We previously confirmed the feasibility and patency of the ELANA Heart Bypass in 6-month follow-up in a porcine model.^6^

Notwithstanding the foreseen assets from standardization and simplification, the ELANA anastomotic construction completely differs from the conventional hand-sewn technique. The ELANA anastomosis configures a side-to-side adventitia-adventitia apposition and introduces foreign material in the form of intraluminal titanium pins. In continuation of the previously published study, we evaluate the process of anastomotic construction, healing, and remodeling for the new prototype ELANA Heart Bypass, with regard to the conventional hand-sewn technique.

## Methods

### Study Design

This is a translational, controlled, follow-up study of 37 pigs (Dutch Landrace, female, mean 66 kg). All underwent off-pump left IMA (LIMA) to LAD surgery either with use of the new ELANA Heart Bypass in 21 pigs or the conventional hand-sewn technique in 16 pigs. The pigs were followed for up to 3 months: 4 hours (*n* = 4), 4 days (*n* = 4), 10 days (*n* = 4), 14 days (*n* = 4), 35 days (*n* = 4), and 90 days (*n* = 17). The specific evaluations performed in the different follow-up periods are described in Table 1.

**Table 1.** Numbers Included in End of Follow-up Analyses.

	ELANA (*n* = 24)			Hand-sewn (*n* = 18)		
Follow-up	CAG	SEM	Histology	CAG	SEM	Histology
4 hours	3	1	1^1^	3	1	1^1^
4 days	2	1	1^1^	2	1	1^1^
10 days	2	1	1^1^	2	1	1^1^
14 days	2	1	1^1^	2	1	1^1^
35 days	2	1	1^1^	2	1	1^1^
90 days	10	0	10^3^	6	0	6^3^
Deceased	3	0	3	1	0	1
Exclusions	0	1	0	0	1	0
Total	24	6	18^8^	18	6	12^8^

Abbreviations: CAG, coronary angiography; SEM, scanning electron microscopy.

Values presented as frequencies, and numbers in superscript are also included in the inflammatory response analyses.

Pigs were excluded from scanning electron microscopy (SEM) in case of complications during the anastomosis construction that could affect anastomotic remodeling. Hence, only *lege artis* anastomoses were evaluated by SEM. Pigs were excluded from histology evaluations on intima hyperplasia and anastomotic size in case of nonpatency. In case of non-device-related mortality or exclusion, the original sample (*N* = 37) was complemented to secure the initially proposed sample size for histology and SEM evaluations. The follow-up period of the new pig corresponds to the follow-up period to the pigs that were excluded or deceased. All animals received care in compliance with the “Guide for the Care and Use of Laboratory Animals,” prepared by the Institute of Laboratory Animal Resources, National Research Council. The animal experimentation committee approved the study protocol.

### Primary Endpoint

The primary endpoint of this study was anastomotic remodeling and neointima formation at the end of the various follow-up periods. SEM was performed for the visualization of neointima formation on the initial nonendothelial surface. Histology evaluations were performed to evaluate anastomotic remodeling, including the amount of neointima formation and/or the formation of intima hyperplasia, the anastomotic opening size, and the inflammatory cell response.

### Secondary Endpoints

The secondary endpoints were patency at the end of follow-up, complication and mortality rates, and perioperative functionality parameters including procedure time, hemostasis, arteriotomy success, and transit time flow measurements (VeriQ Console, Medistim, Oslo, Norway).

### Surgical Procedure

Surgery, anastomotic construction, transit time flow measurements, and coronary angiography were performed according to our previously published protocol and article.^5,6^ The ELANA Heart Bypass was constructed in compliance with its instructions for use, creating a 0-degree side-to-side anastomosis (i.e., functional end-to-side). The hand-sewn anastomoses were constructed 0-degree end-to-side. A running suture (8-0 Prolene, Ethicon Inc, Somerville, NJ) was used over an intracoronary shunt (Medtronic Inc., Dublin, Ireland).

### Tissue Sampling

At the end of follow-up, euthanasia was performed by the administration of pentobarbital sodium (200 mg/kg) intravenously under complete heparinization. This was followed by exsanguination and en bloc excision of the anastomosis. The anastomosis was thereafter fixed overnight with 4% buffered formalin (introduced via the LIMA at 80 mmHg for a minimum of 120 min). The posterior walls of both LIMA and LAD were longitudinally opened for direct inspection (10× to 20× magnification) of ELANA Heart Clip positioning, anastomotic patency, and detection of eventual pseudoaneurysm formation or erosion damage.

### Scanning Electron Microscopy

The anastomoses were fixed, after the perfused-fixation described above, in 2% glutaraldehyde solution buffered in 0.1 M purified phosphate buffer. The fixation was completed by 1-hour 1% buffered osmium tetroxide bathing. Following fixation, the anastomoses were dehydrated in graded series of ethanol (50% to 100%) and liquid CO_2_, using the critical point method. The formation of neointima and eventual thrombocyte coverage of the anastomoses was evaluated using SEM (Philips XL30LAB, FEI Europe, Eindhoven, The Netherlands).

### Histology

The hand-sewn anastomoses were embedded in paraffin (sectioned at 3 to 10 µm transverse slides); the ELANA anastomoses were embedded in methyl methacrylate (sectioned in 350 µm transverse slides). All slides were hematoxylin and eosin stained. The anastomotic dimensions and amount of intima hyperplasia were assessed using AnalySis (Soft-Imaging Software GmbH, Münster, Germany). In a subgroup of the anastomoses, including all follow-up groups, (*n* = 8 ELANA, *n* = 8 hand-sewn; presented in Table 1 in superscript) inflammatory response was determined by counting well-defined nuclei in 6 predefined areas (containing 24 fields) under 400× magnification (fluorescent microscope, Olympus DP71 U-TVIX) with use of the Fiji software.^10^

### Coronary Angiography

Patency was determined at the end of follow-up, per coronary angiography (Philips Allura Xper FD20, Eindhoven, The Netherlands). Access was via the femoral artery, with the internal mammary catheter (6 Fr) introduced in the proximal LIMA. Images were recorded in at least 2 directions and were graded by 2 independent investigators to the FitzGibbon criteria (classes A and B are defined as patent, class O is defined as nonpatent).^11^

### Statistical Analyses

Data were managed in Excel 2010 (Microsoft, Redmond, WA, USA) and analyzed in IBM SPSS Statistics, Version 26.0 (IBM Corp., Armonk, NY, USA). Results are presented in frequency (%) or median (minimum–maximum). Nonparametric tests were performed to compare the baseline and perioperative data between hand-sewn and ELANA anastomoses. Continuous data were compared with use of the Mann-Whitney *U* test, and categorical data were compared with use of Fisher's exact test. Histology measurements (i.e., anastomotic dimension and amount of intima hyperplasia) were compared with use of the Mann-Whitney *U* test. Patency was compared between both groups with use of Fisher's exact test. Throughout, *P* < 0.05 was considered significant.

## Results

**Fig. 1.**
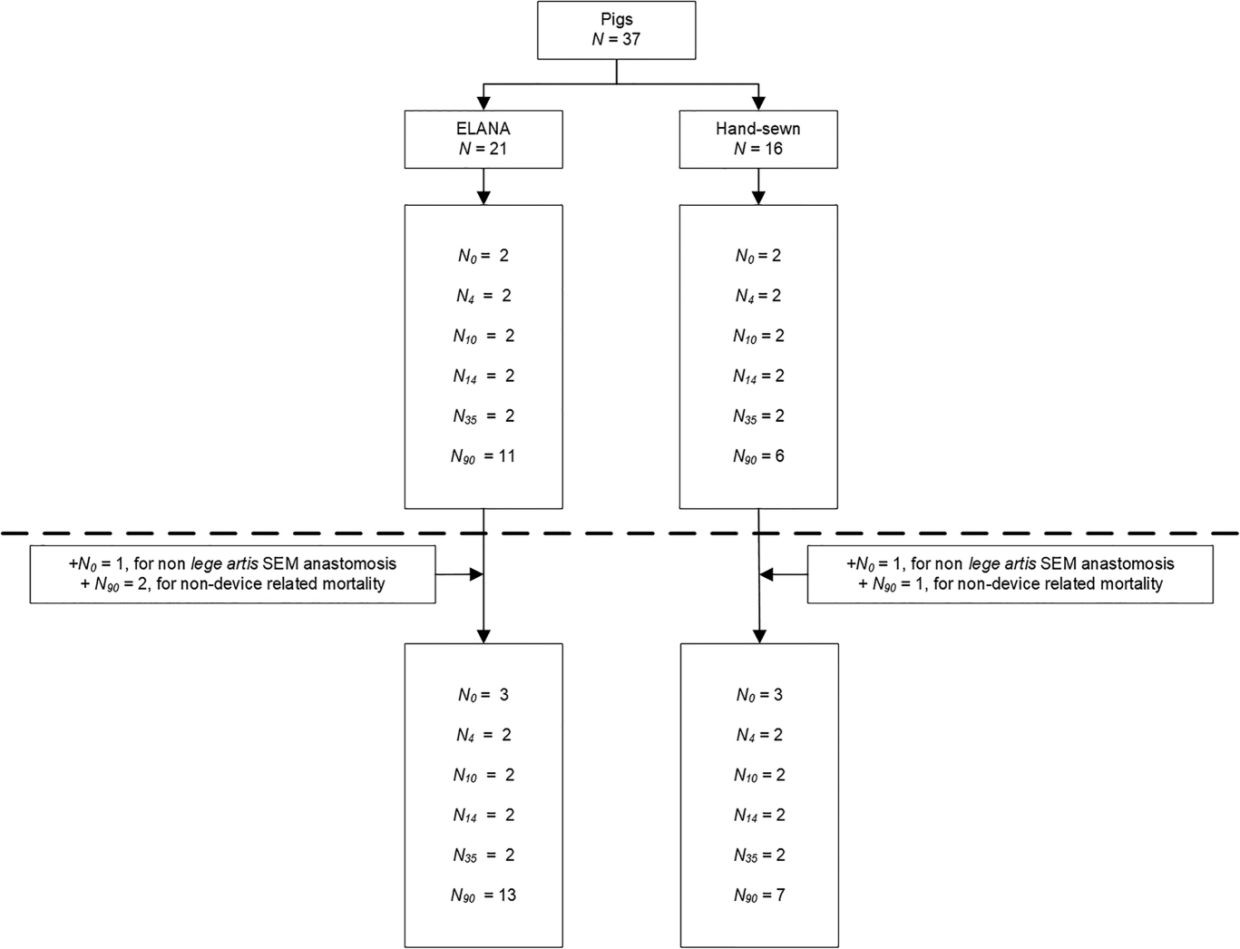
Flowchart of inclusions. The original sample is projected above and the complete sample below the dotted line. Follow-up in days shown in subscript.

Figure 1 presents the total number of pigs included. All 42 pigs were included in follow-up and patency evaluations. One pig in the ELANA group was excluded from SEM analysis for a device-related lesion at the posterior wall during anastomotic construction. One pig in the hand-sewn group was excluded from SEM analysis since it crossed over from the ELANA group following device-related leakage. Four pigs deceased during follow-up; 3 were operated with the ELANA Heart Bypass. One died from respiratory insufficiency (hand-sewn group), and 2 pigs died from unknown reasons (ELANA group). Postmortem examination revealed patent anastomoses in 3 pigs. In the fourth pig a pericardial tamponade and total occlusion of the anastomosis was found (ELANA group). In line with the description above, the original sample of 37 was complemented with 5 pigs.

**Fig. 2.**
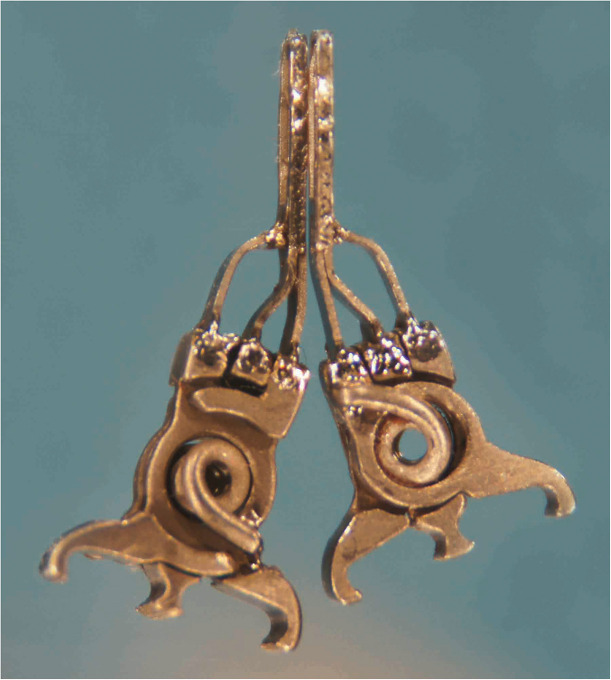
The 2 different ELANA Heart Clip prototypes. The improved spring body of the V3 prototype ELANA Heart Clip (left) enables optimal delivery of spring force onto the pins of the clip. This design change results in a larger and 20% heavier V3 prototype (left) compared with the previous V2 prototype (right).

Out of the 24 ELANA anastomoses, 4 were constructed with the V2 prototype and 20 with the newer V3 prototype ELANA Heart Clip. The V3 prototype was designed to overcome the anastomotic leakage that was evident in the V2 prototype and was directly introduced during this trial. The difference in design between both prototypes is presented in Figure 2.

### Scanning Electron Microscopy

**Fig. 3.**
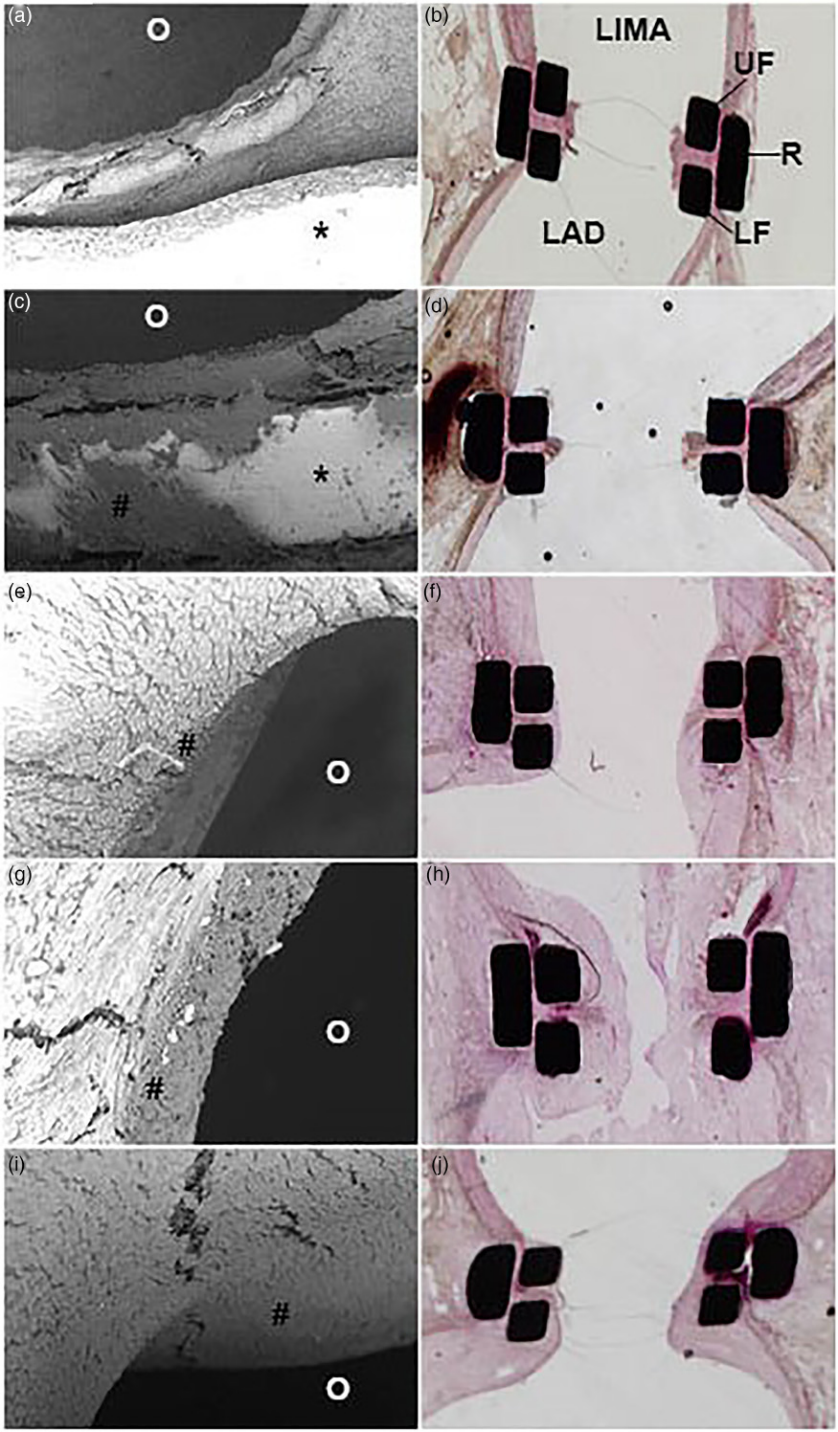
Healing tendency of the ELANA Heart Bypass anastomosis. SEM (left) and light microscopy (right) images of the mid-anastomotic joining line at various follow-up durations. SEM images depict part of the anastomotic rim and titanium pins (*) at shorter follow-up and neointima at longer follow-up durations (#). The anastomotic opening is dark colored (O). Light microscopy images depict transverse sections, mid-anastomosis. The titanium clip parts are black, the squares are transected upper and lower forks (UF and LF), and the rectangles are transected mid-ring (R). LIMA is depicted above and LAD below. The black dots and thin horizontal are artifacts. (a, b) 4 hours postoperative; (c, d) 4 days postoperative; (e, f) 10 days postoperative; (g, h) 14 days postoperative; (i, j) 35 days postoperative. LAD, left anterior descending artery; LIMA, left internal mammary artery; SEM, scanning electron microscopy.

Images of the SEM analyses are presented in Figure 3 (left panel). Four hours postoperative, the ELANA anastomosis showed a sharp laser-cut rim of both LIMA and LAD (Fig. 3a). Limited platelets were attached to the rim and the titanium pins. No endothelial laser damage was detected. At 10 days postoperative, most of the originally nonendothelial surface of the rim and titanium pins was covered by a smooth layer of neointima (Fig. 3e). A continuous endothelial surface from the LAD, over the titanium pins and the rim, into the LIMA was seen at 14 and 35 days postoperative (Fig. 3g, Fig. 3i). In the hand-sewn anastomoses, full endothelial coverage of the Prolene sutures was seen from 10 days onwards.

### Histology

All ELANA anastomoses showed an inverted adventitia-adventitia apposition, whereas the vast majority of the hand-sewn anastomoses showed an everted intima-intima apposition. None of the anastomoses showed pseudoaneurysm formation or erosion damage. Histology images are depicted in Figure 3 (right panel). The ELANA Heart Bypass anastomosis at 4 hours postoperative showed no acute laser damage, some pyknosis of smooth muscle cell nuclei at the compression site, and minor deposition of platelets at the laser rim (Fig. 3b). At 4 days postoperative, the laser rim was covered by a small platelet-rich thrombus (Fig. 3d). From 10 days onwards, the laser rim was retracted and together with the connector pins it was covered with neointima (Fig. 3f). The anastomosis evaluated at 14 days was not patent and covered by a large, organized thrombus (Fig. 3h). At 35 and 90 days, remodeling and neointima hyperplasia were detected (Fig. 3j). Healing in the hand-sewn anastomoses was comparable, with neointima coverage from 10 days and remodeling and neointima hyperplasia seen at 35 and 90 days.

**Fig. 4.**
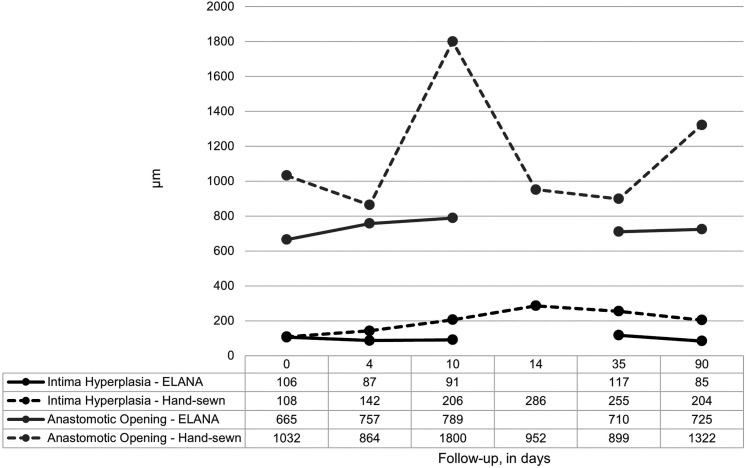
Intima hyperplasia and mid-anastomotic diameter at the end of follow-up. Measurements (in µm, mid-anastomosis, H&E stained slides) are depicted per follow-up term and represent *n* = 1 pig for the follow-up terms up to 35 days and a mean (*n* = 5–8 pigs) for the 90-day follow-up. No data are available for the ELANA 14-day follow-up since the anastomosis analyzed was not patent.

The amount of intima hyperplasia was significantly more in the hand-sewn than in the ELANA anastomoses at 90-day follow-up (229 [150–296] vs 81 [48–1358] µm, *P* = 0.043). Nevertheless, the anastomotic opening was larger in the hand-sewn than in the ELANA anastomoses at 90-day follow-up (1263 [662–1876] vs 780 [433–830] µm, *P* = 0.045). The latter was already preexistent at initial construction. The amount of intima hyperplasia and anastomotic opening size are presented in Figure 4, per specific follow-up duration. Whereas the ELANA anastomotic opening is constant, the variation in hand-sewn anastomotic opening represents the variation in anastomotic construction.

**Fig. 5.**
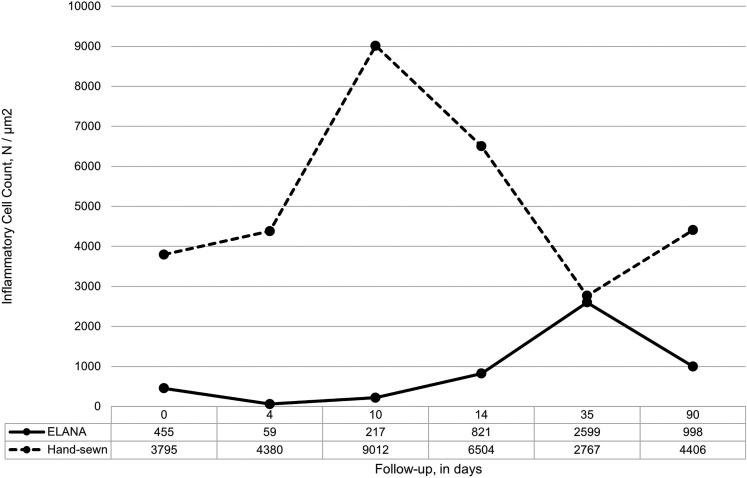
Inflammatory cell density at the end of follow-up. Count of well-defined nuclei in 6 predefined areas (24 fields) near the mid-anastomotic joining line in H&E stained slides (400× magnification). Measurements are depicted per follow-up term and represent *n* = 1 pig for the follow-up terms up to 35 days and a mean (*n* = 3 pigs) for the 90-day follow-up.

Figure 5 presents the inflammatory cell density (well-defined nuclei), which appeared to be about 6 times lower in the ELANA anastomoses (638 [59–2599] vs 4087 [2614–9012] cells/µm^2^, *P* < 0.001). The high cell count in the hand-sewn anastomosis is mainly based on infiltrates detected near the suture material. Specifically in the early follow-up samples (4 hours to 10 days postoperative) acute inflammatory response was apparent. Also, in the ELANA anastomoses inflammatory cells were mainly present near the foreign titanium material.

### Surgery

ELANA and hand-sewn anastomoses were constructed on a target LAD of 1.8 (1.6–2.2) mm and 1.7 (1.6–2.1) mm inner diameter, respectively (*P* = 0.970). Both anastomotic techniques seemed feasible with adequate transit time flow measurements directly and at 1 hour after anastomotic construction, as presented in Table 2.

**Table 2.** Perioperative Characteristics.

	ELANA (*n* =24)	Hand-sewn (*n* = 18)	*P* value
LAD inner diameter, mm	1.8 (1.6–2.2)	1.7 (1.6–2.1)	0.970
LIMA outer diameter, mm	3.0 (2.0–3.5)	3.0 (2.5–3.8)	0.588
Arteriotomy success rate^a^	23 (95.8)	—	
Leakage^b^	1 (4.2)	4 (22.2)	0.146
Construction time, min	3.2 (2.4–4.5)	35.3 (29.7–48.9)	<0.001
Redo^c^	2 (8.3)	0	
Crossover^c^	1 (4.2)	—	
Graft flow at 10 min, mL/min	21.0 (15.0–31.0)	23.0 (17.0–34.0)	0.430
Pulsatility index at 10 min	2.3 (1.5–3.4)	1.9 (1.1–3.8)	0.414
MAP at 10 min, mmHg	91.5 (74.0–114.0)	97.0 (57.0–122.0)	0.169
Graft flow at 60 min, mL/min	23.5 (16.0–37.0)	20.0 (15.0–28.0)	0.139
Pulsatility index at 60 min	2.4 (1.1–4.4)	2.1 (1.4–4.9)	0.672
MAP at 60 min, mmHg	86.5 (70.0–120.0)	89.0 (56.0–116.0)	0.664
Peak hyperemic graft flow, mL/min	84.5 (16.0–138.0)	103.0 (19.5–147.0)	0.253
Hyperemic graft flow response ratio	4.0 (1.0–6.2)	4.6 (1.0–6.8)	0.501

Abbreviations: LAD, left anterior descending artery; LIMA, left internal mammary artery; MAP, mean arterial pressure.

Values presented as *n* (%) or median (minimum–maximum).

^a^ Arteriotomy is stated successful after retrieval of the LAD laser-punched tissue flap using excimer laser.

^b^ Leakage is counted when not self-limiting.

^c^ One redo procedure due to arteriotomy failure (V2 prototype), 1 redo procedure due to nonsolvable leakage (V3 prototype); one crossover from ELANA Heart Bypass to hand-sewn following nonsolvable leakage.

The anastomotic construction time was significantly faster in the ELANA than in the hand-sewn procedures (3.2 vs 35.3 min, *P* < 0.001). Leakage, not self-limiting but generally solved with a single stitch, was found in 2 (8.3%) of the ELANA (2 of 4 V2 prototype, 0 of 20 V3 prototype) and 4 (22.2%) of the hand-sewn anastomoses (*P* = 0.375). Apart from the above, one ELANA Heart Bypass anastomosis needed reconstruction for leakage (V3 prototype), and another for arteriotomy failure secondary to a malfunctioning fixation device (V2 prototype). The first anastomosis was redone with suture and crossed over since no additional ELANA devices were available at that moment; the second was successfully redone with a new ELANA Heart Clip. None of the hand-sewn anastomoses needed reconstruction. No mortality occurred during surgery.

### Patency

At the end of follow-up, coronary angiography images determined 18 of 18 hand-sewn and 19 of 24 ELANA Heart Bypass anastomoses were patent (100% vs 79%, *P* = 0.06). One of the occlusions was found in a pig deceased before the end of follow-up, as was previously described. The other occlusions were found at the end of follow-up (1 at 14 days, 3 at 90 days). This was despite initial uncomplicated ELANA Heart Bypass procedures.

## Discussion

Our recent study demonstrated preclinical feasibility of the ELANA Heart Bypass technique (V2 prototype) up to 6 months of follow-up; however, the anastomotic leakage rate after construction was unacceptable.^6^ The current study supports technical perioperative feasibility of the new V3 prototype and a reduced rate of anastomotic leakage. More important, the anastomotic remodeling was evaluated with regard to the conventional hand-sewn anastomosis. It appeared that the initial non-intima surfaces of both the ELANA and hand-sewn anastomoses were covered with neointima from 14 days postoperative. Less intima hyperplasia developed in the preexistent smaller ELANA anastomosis, and a lower inflammatory response was detected. Yet the ELANA technique tends to result in a lower patency rate compared with the hand-sewn anastomoses.

Whereas sutureless anastomotic devices could boost minimally invasive coronary surgery, several properties have to be evaluated before a new facilitated anastomotic technique can be implemented in clinical practice. Most criticized is the non-intima surface inside the anastomosis, created by implantation of foreign material and alternative unconventional vessel wall apposition. In contrast, the vessel wall in hand-sewn anastomoses is everted in an intima-intima apposition, with a continuous monofilament wire introduced in about 12 stitches. Scheltes et al. introduced the term “blood-exposed non-intimal surface” (BENIS) to compare the variety of anastomotic construction techniques. It was hypothesized that an increased BENIS could increase the risk on thrombosis, intima hyperplasia, and stenosis in the longer term.^12^ The BENIS of the ELANA Heart Bypass is indeed larger than the hand-sewn technique (8.11 vs 1.3 mm^2^). This results from the adventitia-adventitia positioning, exposing media and adventitia, and the intraluminal positioning of titanium pins in both the graft and coronary artery. However, our current study determined less intima hyperplasia inside the ELANA anastomosis compared with the hand-sewn anastomoses. Consequently, this finding suggests that the initial anastomotic opening created by excimer laser will sustain. The latter was also described in evaluations of other distal anastomotic devices. Suyker et al. described limited lumen loss by less intima hyperplasia in their S2-connector with stainless steel staples compared to Prolene suture in a porcine model.^13^ Also for the C-port with stainless steel staples and the nitinol U-clip no additional intima hyperplasia was described, and remodeling was defined comparable to the hand-sewn technique.^14,15^ Hence, BENIS seems not the only explanatory factor for the amount of intima hyperplasia. However, devices with even larger BENIS have encountered less optimal clinical results in the longer term.^16^

There are several mechanisms that might have reduced the amount of intima hyperplasia in the ELANA anastomoses. The ELANA Heart Bypass technique is designed to limit tissue trauma due to a single puncture of the vessel wall in contrast to the graft and coronary artery manipulation needed for a running suture. Thus, the Prolene sutures apply direct tissue compression, whereas the compression force of the ELANA Heart Clip is distributed via the full length of the pins. Notwithstanding the above, we should mention that the anastomotic opening of the ELANA was smaller than the opening of the hand-sewn anastomosis. An acceleration of flow through the smaller ELANA anastomosis might bias the onset of intima hyperplasia at the anastomotic joining line.

Although histology analyses show favorable results for the ELANA technique with limited intima hyperplasia, patency at the end of follow-up was disappointing compared with the hand-sewn anastomosis (79% vs 100%). Histology analyses of the non-patent ELANA anastomoses show distortion of the anastomotic complex. There is limited evidence that the inferior patency results from displacement and up to 90° tilting of the ELANA Heart Clip. More specifically, the outbalanced weight and length of the ELANA Heart Clip may induce distortion and tilting of the LIMA-LAD complex on the epicardium of the beating heart. Distortion on itself can cause traction on the LAD and consequently increase wall-shear stress and secondary development of intima hyperplasia, or direct occlusion of the compromised lumina. Within this study we measured adequate transit time flow after anastomotic construction. We did not perform interim coronary angiography or intracoronary evaluations before the end of follow-up. Therefore, we cannot guarantee adequate position of the ELANA Heart Clip pins, as we cannot define the moment of anastomotic occlusion. The pins might have been positioned partially intramural rather than completely intraluminal, or intima damage could have occurred during anastomotic construction. Both intima damage and intramural positioning of the pins might induce the development of intima hyperplasia, ultimately resulting in a non-patent anastomosis.

This study was not designed to compare V2 and V3 ELANA Heart Clip prototypes. Notwithstanding this limitation in study design, we do believe that the inferior patency outcomes represent the current ELANA Heart Bypass technique adequately. These results warrant careful examination and urge further innovation. We opt to develop and evaluate a less bulky, smaller, and lighter prototype ELANA Heart Clip to prevent distortion and tilting of the facilitated anastomosis. Together with the current information on anastomotic remodeling, this renewed preclinical evaluation could be the key toward optimal design of the sutureless coronary anastomosis.

## Conclusions

This study shows the technical perioperative feasibility of the ELANA Heart Bypass System. Although limited intima hyperplasia was observed, hand-sewn anastomoses had superior patency during follow-up. An additional study with a new prototype is required before clinical implementation.

## Supplemental Material

Visual abstract - Supplemental material for Preclinical Comparison of Distal Off-Pump Anastomotic Remodeling: Hand-Sewn Versus ELANA Heart Bypass Click here for additional data file. Supplemental material, sj-pptx-1-inv-10.1177_15569845221079606 for Preclinical Comparison of Distal Off-Pump Anastomotic Remodeling: Hand-Sewn Versus ELANA Heart Bypass by David Stecher, Marieke Hoogewerf, Bart P. van Putte, Shadan Osman, Pieter A. Doevendans, Cornelis Tulleken, Lex van Herwerden, Gerard Pasterkamp and Marc P. Buijsrogge in Innovations
