# Supplementary material for: Preclinical Comparison of Distal Off-Pump Anastomotic Remodeling: Hand-Sewn Versus ELANA Heart Bypass
Source: Innovations (Phila). 2022 Mar 17;17(2):111–8. doi: 10.1177/15569845221079606 (PMC9066685; doi:10.1177/15569845221079606)
Supplement: Visual abstract - Supplemental material for Preclinical Comparison of Distal Off-Pump Anastomotic Remodeling: Hand-Sewn Versus ELANA Heart Bypass [file sj-pptx-1-inv-10.1177_15569845221079606.pptx]

## Slide 1
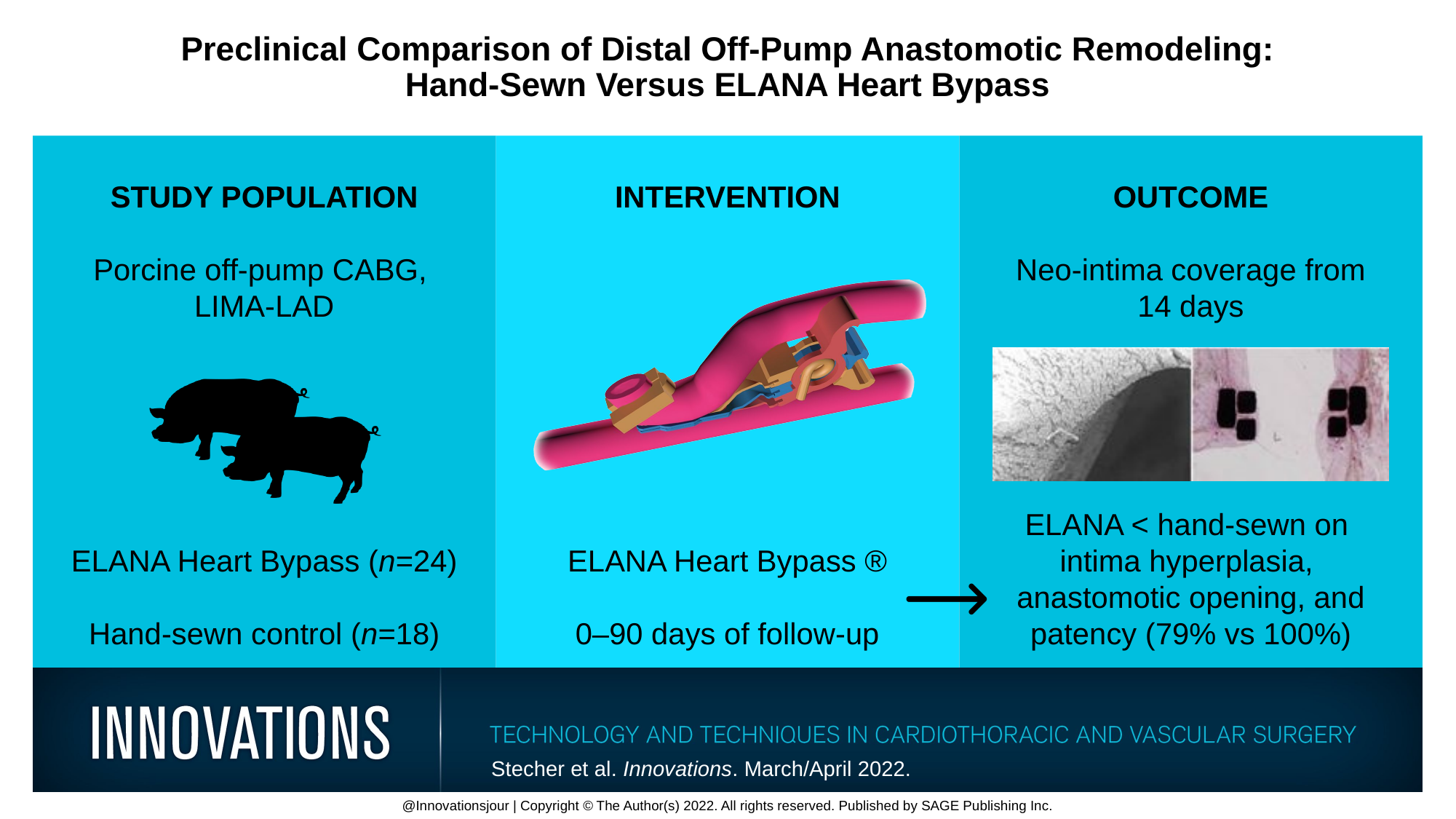

# Preclinical Comparison of Distal Off-Pump Anastomotic Remodeling: Hand-Sewn Versus ELANA Heart Bypass
STUDY POPULATION
Porcine off-pump CABG,
LIMA-LAD
ELANA Heart Bypass (n=24)
Hand-sewn control (n=18)
INTERVENTION
ELANA Heart Bypass ®
0–90 days of follow-up
OUTCOME
Neo-intima coverage from
14 days
ELANA < hand-sewn on
intima hyperplasia,
anastomotic opening, and patency (79% vs 100%)
Stecher et al. Innovations. March/April 2022.
@Innovationsjour | Copyright © The Author(s) 2022. All rights reserved. Published by SAGE Publishing Inc.
